# Revised Morning Loops of the *Arabidopsis* Circadian Clock Based on Analyses of Direct Regulatory Interactions

**DOI:** 10.1371/journal.pone.0143943

**Published:** 2015-12-01

**Authors:** Sally Adams, Ian Manfield, Peter Stockley, Isabelle A. Carré

**Affiliations:** 1 School of Life Sciences, University of Warwick, Coventry, United Kingdom; 2 Astbury Centre, University of Leeds, Leeds, United Kingdom; Karlsruhe Institute of Technology, GERMANY

## Abstract

The network structure of the plant circadian clock is complex and direct regulatory interactions between individual components have proven particularly difficult to predict from genetic analyses. Here, we systematically investigate in vivo binding interactions between the morning-specific transcription factor, LATE ELONGATED HYPOCOTYL (LHY) and the promoters of other components of the network. We then demonstrate the functionality of these interactions by testing the responsiveness of the target gene to an ethanol-induced change in expression level of the LHY protein. We uncover novel, negative autoregulatory feedback loops from *LHY* and the closely related *CIRCADIAN CLOCK ASSOCIATED-1 (CCA1)* onto their own and each other’s expression. Furthermore we show that LHY acts as a repressor of all other clock components, including *PSEUDO-RESPONSE REGULATORs* (*PRRs*) 9 and 7, which were previously thought to be positive regulatory targets. These experimental results lead to a substantial revision of the morning loops of the clock.

## Introduction

One fascinating aspect of Biology is the ability of most organisms to keep time and to anticipate predictable changes in environmental conditions. Daily rhythms, controlled by an endogenous circadian clock, have been identified in a wide range of organisms ranging from cyanobacteria to plants, fungi and mammals. The molecular mechanism of these clocks has been extensively studied over the past 20 years, and was shown to be largely based on networks of negative, interlocked transcriptional-translational feedback loops, where positive and negative components regulate each other’s expression to generate approximately 24 hour oscillations [1].

The plant circadian clock is composed of a set of proteins distinct from its animal and fungal counterparts. Recent work suggested that its oscillatory mechanism is also distinct in its architecture, in that its core feedback loop is composed of three inhibitory steps [2–4]. The two MYB transcription factors, LATE ELONGATED HYPOCOTYL (LHY) and CIRCADIAN CLOCK ASSOCIATED-1 (CCA1) peak in the morning, and act to repress expression of a pseudo response regulator (PRR1, also known as TIMING OF CAB-1, or TOC1) during the day, by binding to an Evening Element (EE) motif in the promoter of its gene. As LHY/CCA1 protein levels decline towards the evening, TOC1 accumulates and acts to repress transcription from their respective promoters. *TOC1* transcription is then down-regulated late at night by an Evening Complex (EC) composed of three proteins, LUX and EARLY FLOWERING (ELF) 3 and 4 and this enables transcription of *LHY* and *CCA1* to resume at the following dawn.

Additional feedback loops are mediated by three other PRR proteins, PRR 9, 7 and 5. These proteins are expressed in sequential waves throughout the day [5], and bind to the *LHY* and *CCA1* promoters to repress their activity. Altogether, the PRR proteins and TOC1 ensure that expression of *LHY* and *CCA1* is repressed from the late morning until the following dawn [6]. Recent work also identified a number of rhythmically expressed transcriptional activators that also contribute to the function of clock. REVEILLE (RVE) 4, 6 and 8 up-regulate the afternoon and evening specific genes *PRR5*, *TOC1*, *GI*, *ELF4* and *LUX* as well as the morning-specific *PRR9*; The Light-regulated WD1 (LWD1) and LWD2 proteins activate the expression of *PRR9*, *PRR5* and *TOC1*, and the LNK transcription factors 1 and 2, the expression of *PRR5* and *ELF4* [7–11].

Genetic methods can prove unreliable when investigating regulatory interactions as part of highly interconnected gene networks such as the plant circadian clock [3]. In order to further investigate the structure of this network, we tested the direct binding of LHY and CCA1 to genes encoding other oscillator components. We then confirmed the regulatory function of these physical interactions.

## Results and Discussion

### LHY binds to the promoter of all clock genes including itself and CCA1

**Fig 1:**
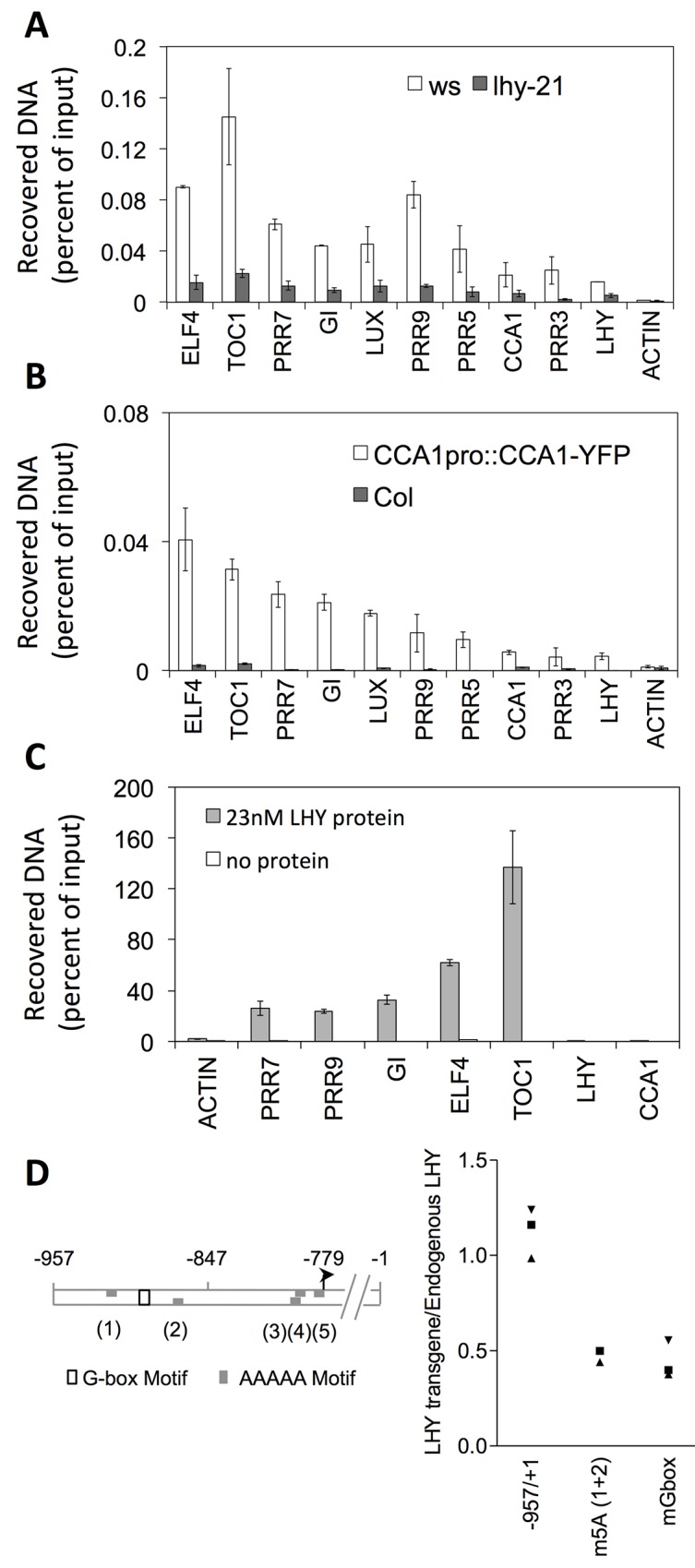
Binding of LHY and CCA1 to the promoters of clock-associated genes. **(A)** In vivo binding of LHY to the promoters of clock-associated genes was tested by ChIP-qPCR analyses of wild-type samples using a polyclonal antibody to the LHY protein. Plants were grown under 12L12D light-dark cycles and tissue was sampled two hours after dawn, corresponding to the time when LHY protein levels are at their maximum [31]. The *ACTIN* locus was used as a negative control. In order to demonstrate the specificity of the antibody, ChIP enrichments for wild-type (Ws) plants were compared to those for the insertion mutant, *lhy-21*. Error bars represent standard errors from two independent biological experiments. **(B)** In vivo binding of CCA1 was tested by performing ChIP-qPCR analyses on *cca1-1 CCA1pro*::CCA1-HA-YFP or wild-type plants using an antibody to the YFP tag. Enrichment for the *ACTIN2-7* locus is shown as a negative control. Error bars represent standard errors from two independent biological experiments. **(C)** In vitro binding of LHY to purified genomic DNA. Bacterially expressed, His-tagged LHY protein was used to pull down sheared, purified genomic DNA. The resulting enrichment for different target promoter sequences was quantified by real time PCR using the same primers as used for ChIP analyses, and expressed as a percentage of input material. This experiment detected binding to all LHY binding targets identified in ChIP experiments, except for the *LHY* and *CCA1* promoters. Error bars represent standard deviations from two independent binding experiments. **(D)** Mutations of the G-box and 5A sites disrupt in vivo binding of the LHY protein to its own promoter. The diagram at the top of the panel shows the relative positions of G-box and 5A motifs in the LHY promoter. Positions are numbered relative to the ATG and the arrow represents the transcriptional start site. ChIP experiments were carried out to assay binding of the LHY protein to wild-type and mutated *LHY*::*luc* transgenes. In order to account for differences in sample preparation, enrichment for *LHY*::*luc* transgene sequence was expressed relative to endogenous *LHY* promoter sequence. Relative enrichment levels close to 1 were obtained using the wild-type construct (-957/+1), indicating equivalent enrichment for the endogenous and the transgenic copies of the *LHY* promoter. However, mutation of the G-box flanking regions (ACCACGTGTC to GTCACGTGAC) reduced binding of the LHY protein by over 50%. Mutation of both flanking 5A sites [5A (1) CCAAAAA to TGTCAAA and 5A(2) TTTTTCC to TTTGACA] had a similar effect. Each data point represents the relative enrichment for one transgenic line. Error bars from Q-PCR analyses have been omitted for clarity.

We investigated the binding of LHY to individual promoters in chromatin immunoprecipitation (ChIP) experiments. This confirmed known interactions with the promoter of *TOC1* [12, 13], *PRR7* and *9* [14], *ELF4* [15], *ELF3*, *GI* [16] and *LUX* [17] and verified interactions with the *PRR3*, *PRR5* and *CCA1* promoters that were predicted based on the presence of EE or EE-like motifs (Fig 1A). Similar ChIP analyses using *cca1-1 CCA1pro*::CCA1-HA-YFP plants [18] and an antibody to the YFP tag showed that LHY and CCA1 have similar binding preferences (Fig 1B). This suggests that LHY and CCA1 mediate identical regulatory connections as part of the oscillatory mechanism of the clock, and supports the previous suggestion that their function as part of the clock mechanism is largely redundant [19, 20].

The ChIP experiments also identified a novel interaction with an evolutionarily conserved region of the *LHY* promoter [21], which was surprising because no known binding motif was present within this genomic region. To test whether LHY might be recruited to its own promoter via interactions with other DNA-binding proteins, we tested the *in vitro* binding of purified LHY protein to purified genomic DNA. Pull-down experiments using bacterially expressed, His-tagged LHY protein as bait resulted in significant enrichment for all of the binding targets identified Fig 1A, except for the *LHY* and *CCA1* promoters (Fig 1B). This result suggests that binding of LHY to the *LHY* and *CCA1* promoters requires protein cofactors.

Known regulatory motifs within the LHY promoter include a G box and multiple 5A motifs [21]. Disruption of these motifs by site-directed mutagenesis of a *LHY*:*luciferase* (*LHY*:*luc*) reporter construct was previously shown to result in reduced amplitude of the luminescence rhythm in transgenic plants. To test whether either of these motifs might act to recruit LHY to its own promoter, we compared in vivo binding of the LHY protein to wild-type and mutated *LHY*:*luc* reporter constructs in ChIP experiments (Fig 1C). The enrichment level obtained for the wild-type *LHY*:*luc* transgene was similar to that for the endogenous *LHY* promoter, as indicated by a relative enrichment level close to 1. However mutation of the G-box motif in the *LHY*::*luc* construct reduced the enrichment level 2 to 3 fold. Mutation of the two 5A motifs flanking the G-box reduced enrichment to a similar extent. These results suggest that LHY binding to its own promoter is mediated, at least in part, through interactions with G box- and 5A-binding factors. Similar motifs present within the promoter of *CCA1* may also account for LHY binding to that promoter [21].

### LHY represses transcription of all of the *PRR* genes

**Fig 2:**
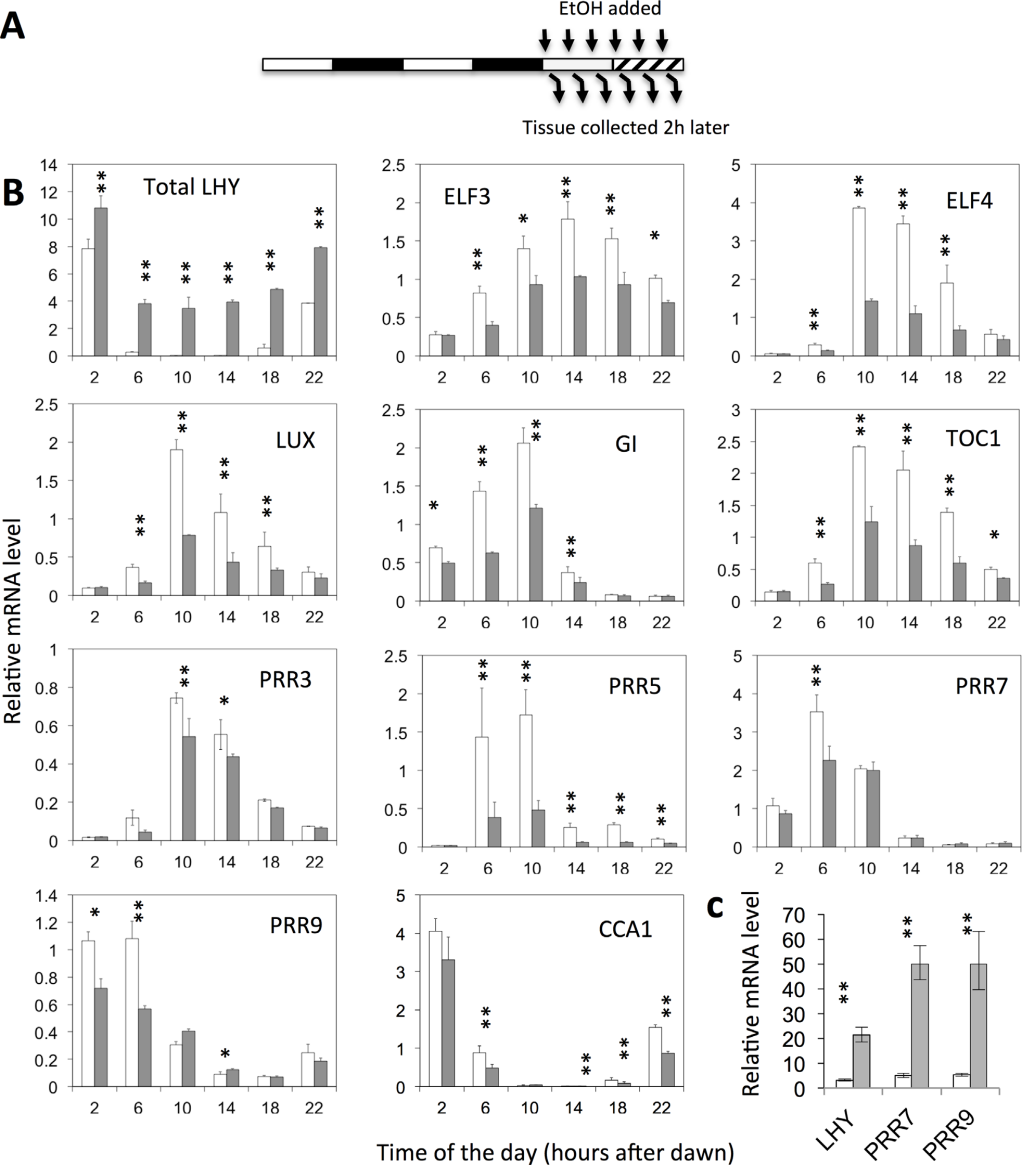
LHY represses expression of other clock components. **(A**) Experimental design. Wild-type and *Alcpro*::*LHY* transgenic plants were grown under 12L12D light-dark cycles as illustrated by the white and black bars in the diagram. They were transferred to constant light at the start of the experiment. Expression of the *Alcpro*::*LHY* transgene was induced using 6% ethanol (v/v). Different sets of plants were treated at 4-hour intervals over the duration of one circadian cycle, and tissue was harvested 2 hours later. (**B**) mRNA levels were determined using Nanostring technology and normalized relative to *UBC12*. Times indicate when the tissue was harvested. **(C)** shows levels of *PRR7* and *PRR9* mRNA expression 26 h after induction of the *Alcpro*::*LHY* transgene at ZT17. Open bars indicate wild-type data (+EtOH), filled bars *Alcpro*::*LHY* data (+EtOH). Transcript levels from Quantitative RT-PCR analyses were normalized relative to *ACTIN*. Data shown are averages and standard errors from two independent biological replicates. * indicates p <0.05 and ** p<0.01 as determined by t-tests. An additional experiment comparing comparing effects of ethanol on *PRR7* and *PRR9* expression in *Alcpro*:::*LHY* plants, *Alcpro*::*GUS* and wild-type plants, 2, 6 and 10 hours after dawn is provided as S1 Fig.

In order to test the regulatory function of promoter-binding interactions, we assayed the rapid changes in mRNA levels that followed induction of an ethanol-responsive *LHY* transgene (*ALCpro*::*LHY*). This experimental design enabled us to circumvent possible artifacts of constitutive overexpression or knock-down experiments, such as indirect effects of LHY that may be mediated by other components of the network. In order to uncover possible time-of-the-day dependency (i.e., gating) of the effect of LHY on its different regulatory targets, similar induction experiments were carried out at 4 hour intervals over the duration of the circadian cycle (Fig 2A).

Induction of the *ALCpro*::*LHY* transgene led to the repression of other components of the network, including *ELF3*, *ELF4*, *LUX*, *GI*, *TOC1*, *PRR3*, *PRR5*, *PRR7*, *PRR9* and *CCA1* (Fig 2B and S1 Fig). Down-regulated transcript levels were observed within 2 hours of ethanol addition, and most pronounced effects were observed at times corresponding to peak transcript levels.

Previous work showed that expression of the *PRR7* and *PRR9* transcripts was elevated during the night in *LHY- and CCA1-overexpressing* (*LHY-ox and CCA1-ox*) plants, and reduced during the day in *cca1-1 lhy-R* double mutants [22]. In agreement with these observations, we found that induction of *Alcpro*::*LHY* expression led to elevated *PRR7* and *PRR9* transcript levels, 26 hours after ethanol addition (Fig 2C). Based on these results, we suggest that LHY acts as a direct repressor of *PRR7* and *PRR9 t*ranscription, and that the elevated expression of these transcripts in LHY-ox plants reflects indirect effects, mediated by feedback.

### LHY and CCA1 repress their own and each other’s expression

**Fig 3:**
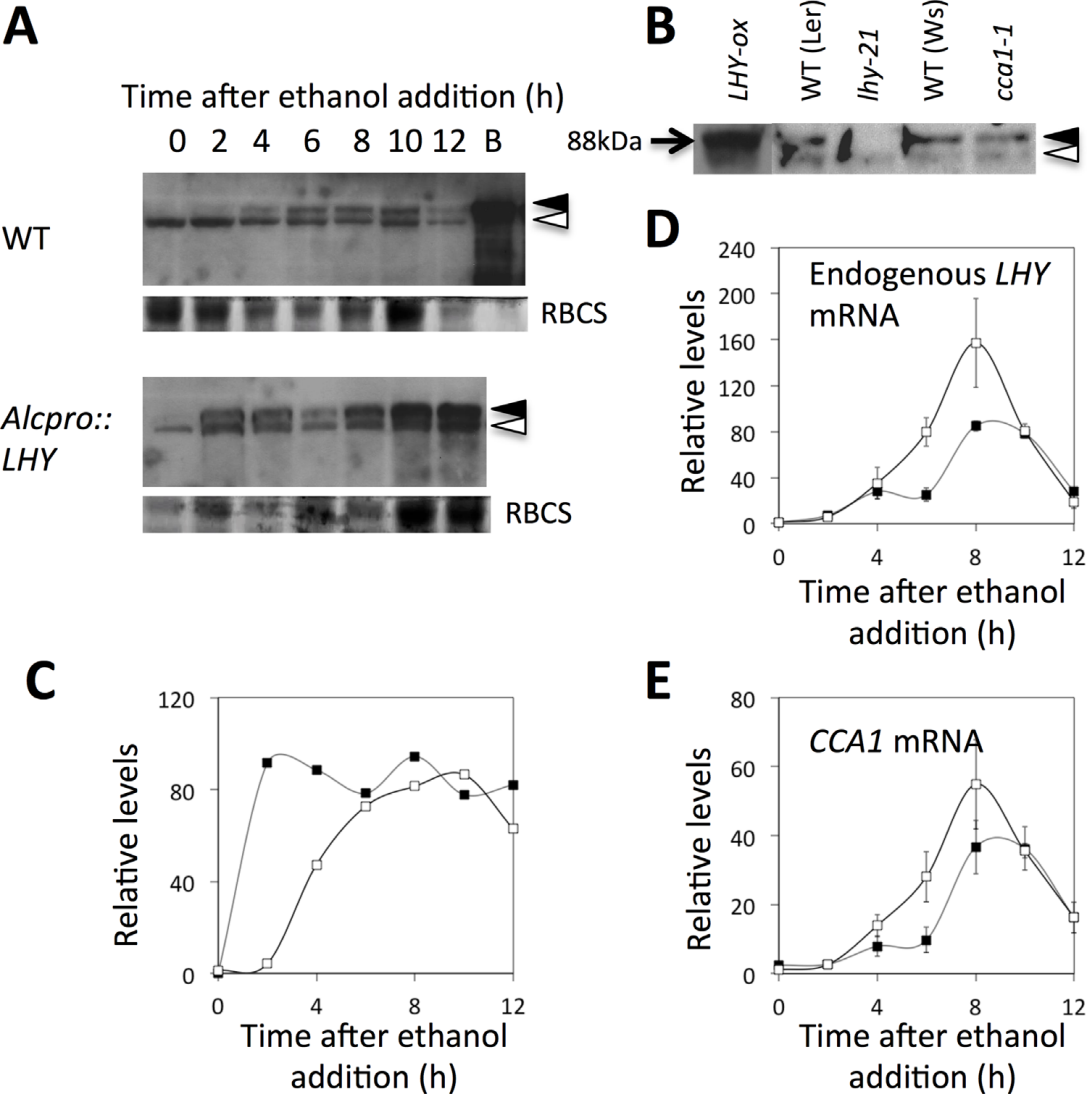
Induction of the *ALCpro*::*LHY* transgene abrogates the peak of *LHY* and *CCA1* expression at dawn. Ethanol (1% v/v) was added to plants 17 hours after dawn, i.e. just before the normal rise in *LHY* transcription. **(A, B)** Immunoblot showing changes in LHY protein levels after ethanol addition, and control experiment showing the specificity of the LHY antibody. The LHY protein is indicated by filled triangles, and a constitutive, cross-reactive band is indicated by open triangles. B indicates bacterially expressed LHY protein. As a loading control, the lower part of the gel was stained with Coomassie blue to reveal the RBCS protein. **(C)** Quantification of LHY protein levels from (A). LHY protein levels were normalized to the cross-reactive band and expressed relative to wild-type levels at time zero. **(D, E)** Changes in endogenous *LHY* and *CCA1* mRNA levels as determined by quantitative RT-PCR. Transcript levels were normalized to the *ACTIN* transcript and to levels in control plants at time zero. Open symbols indicate wild-type and filled symbols, *Alcpro*::*LHY* data. Data shown are averages and standard errors from triplicate quantitative RT-PCR analyses. A replicate experiment in shown in S2A–S2C Fig.

*CCA1* expression was down-regulated rapidly following *Alcpro*::*LHY* induction (Fig 2B). This showed that LHY acts as a direct repressor of *CCA1* transcription. Downregulation of the LHY transcript was also observed following induction of the *Alcpro*::*LHY* transgene in the middle of the subjective night (at ZT17, i.e. 17 hours after dawn) (Fig 3C). Reduced *LHY* transcript levels were observed 4–8 h after ethanol addition (Fig 3C), mirroring effects on CCA1 transcript levels (Fig 3D). Experiments testing the effects of induction of an ethanol-inducible *Alcpro*::*CCA1* transgene produced similar results (S2 Fig). Altogether, these results suggest that, although they don’t bind DNA at their own promoters, both LHY and CCA1 repress of their own and of each other’s transcription by forming physical interactions with other transcription factors.

## Conclusion

**Fig 4:**
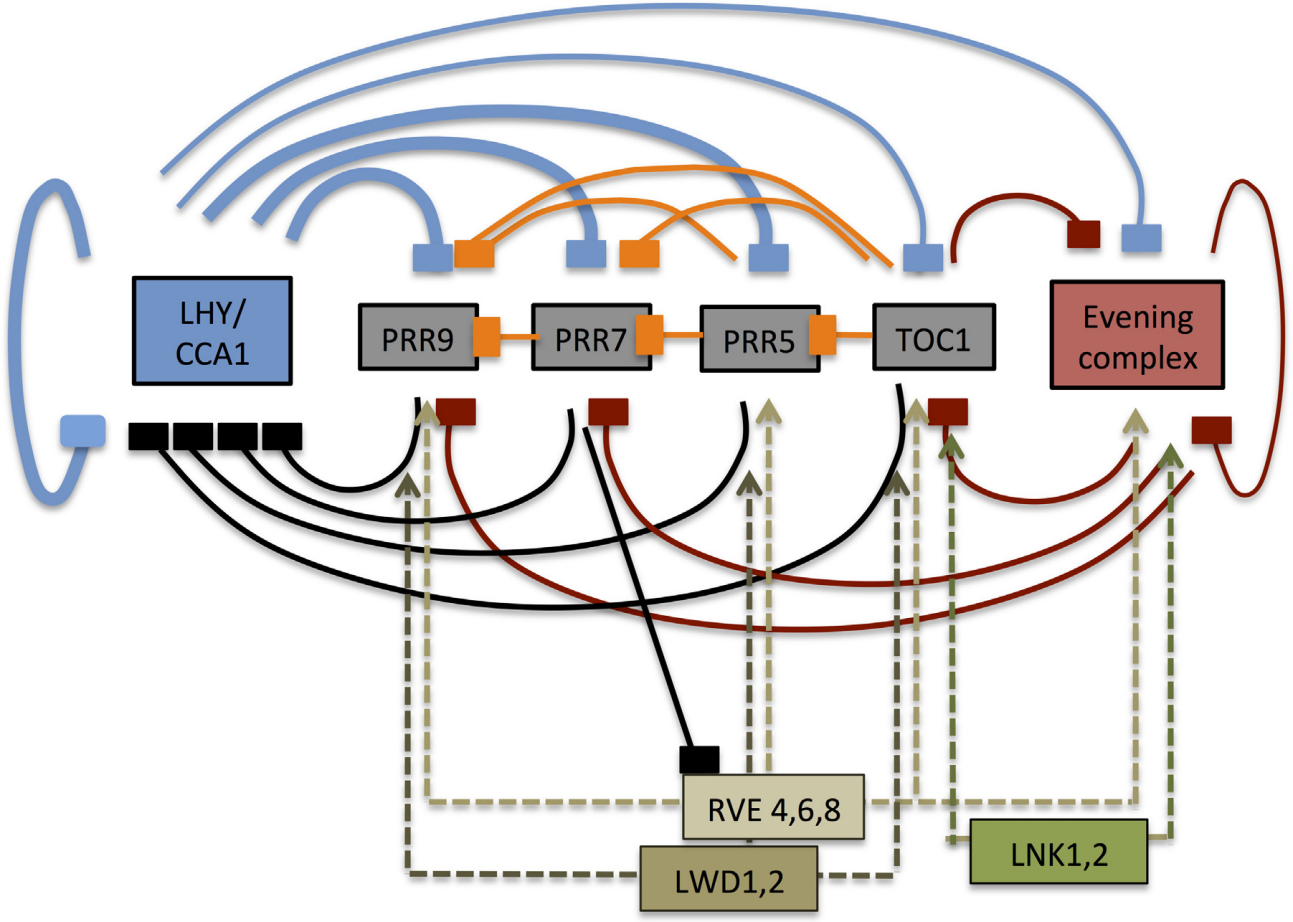
The transcriptional network of the plant circadian clock. Revised regulatory connections arising from this work are indicated by heavier lines. We add a novel, direct autoregulatory loop from *LHY*/*CCA1* onto their own expression and we introduce a change in the sign of the regulation of *PRR5*, *7* and *9* transcription by *LHY* and *CCA1*. Furthermore, we show that the PRR5 gene is also down-regulated by *LHY/CCA1*. Each box indicates a gene. Pointed arrows indicate positive regulation and blunt arrows, transcriptional repression. Blue arrows represent regulation by *LHY*/*CCA1*, red arrows regulation by the evening complex (EC) and black arrows regulation by the *PRRs* and *TOC1*. Orange arrows indicate regulatory interactions between the *PRR* genes, and dashed arrows indicative positive regulation by the RVE, LNK or LWD proteins.

Our experimental results lead to substantial alterations of the morning loops of the plant circadian clock, as summarized in Fig 4.

LHY and CCA1 were until now thought to promote transcription of *PRR7* and *PRR9*. This was based on the observation, that expression of the *PRR7* and *PRR9* transcripts was elevated during the night in *LHY- and CCA1-overexpressing* (*LHY-ox and CCA1-ox*) plants, and reduced during the day in *cca1-1 lhy-R* double mutants [22]. However, our results show that induction of LHY expression causes immediate down-regulation of all of the *PRR* genes, including *PRR9*, *7*, *5* and *PRR1/TOC1*. This demonstrates that LHY functions as a transcriptional repressor of these genes. We propose that the elevated levels of *PRR7* and *PRR9* transcripts in LHY- and CCA1-overexpressing plants reflect long-term, indirect effects due to altered expression of other components of the network.

LHY and CCA1 were previously shown to act as negative regulators of their own expression [23, 24], but the generally accepted model was that this regulation was indirect. LHY and CCA1 are known to function as part of a negative feedback loop, in which their expression is repressed by the PRR proteins and TOC1 during the day, and this repression is lifted when the EC represses *TOC1* transcription late at night [2]. The new finding, that LHY acts as a repressor of all of the *PRR* genes raises an issue with this model, as it implies that LHY switches off expression of all of its inhibitors. This implies that once LHY and CCA1 expression is switched on in the morning, their expression will remain high and the repression of the *PRR* genes will be maintained. Oscillatory behavior will be prevented unless some other mechanism is present, either to shut down expression of *LHY* and *CCA1* or to override their effect on *PRR 9* and *PRR7* transcription. Our finding, that LHY and CCA1 act to directly downregulate their own transcription provides one such mechanism.

This direct autoregulation of *LHY* and *CCA1* and the negative regulation of *PRR7* and *PRR9* expression by LHY were both incorporated into a recent mathematical model for the clock, albeit without experimental justification [25]. This model demonstrates that both of these features are compatible with the temporal patterns of oscillations of the clock genes. However, analyses of the model have so far focused on the role of positive regulators of the PRR genes, also newly incorporated into the model. Further modeling will be required in order to fully understand the functional implications of the revised network architecture. In the revised structure each of the *PRR* genes (including TOC1) is regulated in a highly similar fashion expression by LHY/CCA1 and by the evening complex. This now blurs the functional distinction between the so-called morning loops of the clock, mediated by PRR9 and PRR7, and the evening loops mediated by TOC1 [26]. The similar structure of the *PRR* and *TOC1* feedback loops would suggest redundant functions as part of the oscillatory mechanism of the clock, and yet we know that mutations in these different genes result in distinct period phenotypes [27, 28]. The key to their different functions lies in the differential timing of their expression, with the night-specific TOC1 and PRR5 controlling the onset of *LHY* expression and the morning-specific PRR9 controlling its offset [6]. We still need to develop a better understanding of the mechanism by which the sequential waves of *PRR* gene expression are generated.

## Material and Methods

### Plant material and growth conditions

Wild-type transgenic lines carrying the *LHY*::*LUC*, *ALCpro*::*LHY* and *ALCpro*::*CCA1* constructs and the *cca1-1* mutant line carrying the *CCA1pro*::CCA1-HA-YFP transgene have been described previously [18, 21, 29]. Seeds were sown on MS-agar plates, stratified in the dark for 3 days at 4°C and grown under 12-h photoperiods at 22°C unless otherwise stated.

### Chromatin immunoprecipitation (ChIP)

Chromatin was isolated from 2-week-old seedlings harvested 2 h after dawn. Immunoprecipitation was carried out according to [30] using a polyclonal antibody to the LHY protein previously described by [31]. DNA was eluted from protein A beads in the presence of 10% Chelex according to [32] and analysed by qPCR. Results were expressed relative to the original input chromatin sample. All primers used are listed in S1 Table.

### Gene expression analyses

Total RNA was extracted from seedlings using the Plant RNeasy kit (Qiagen) and contaminating genomic DNA removed by treatment with DNaseI (SIGMA). First-strand cDNA synthesis was carried out using Revert-aid H-Minus M-MuMLV Reverse transcriptase (Fermentas) and primed using random DNA hexamers. Expression levels were determined by qPCR as above and calculated relative to *ACT2* (At3g18780). Alternatively, mRNA expression levels were quantified using Nanostring® technology [33] and expressed relative to *UBC12 (*AT3g08700).

### Ethanol induction of *ALCpro*::*LHY* and *ALCpro*::*CCA1* lines

5 ml of ethanol (1 to 6% v/v) was added directly to the roots of the plants. In order to maintain ethanol vapours, a 3 cm^2^ piece of filter paper soaked in ethanol was placed on the underside of the plate lid at hourly intervals. Expression of the LHY protein following induction was quantified by immunoblot analyses and was calculated relative to a constitutive cross-reacting band.

### LHY protein expression in E. coli

The LHY protein was expressed in E. coli BL21(DE3)pREP4-RIL cells (Stratagene) as a C-terminal hexa-histidine fusion. It was purified by chromatography on HisTrap FF column (GE Healthcare) then on a HiTrap Q HP column (GE Healthcare).

### Genomic DNA pull-down

*Arabidopsis* genomic DNA was isolated using the PHYTOPURE™ extraction kit (GE healthcare), sonicated to generate fragments of 100 to 600 bp2, then incubated with purified, recombinant LHY:His protein for 2 hours at 20°C. DNA-protein complexes were pulled-down using Ni-NTA magnetic beads (Dynabeads). DNA was purified using the MinElute PCR purification kit (Qiagen) prior to q-PCR analyses. The same primer sets were used as in ChIP analyses (S1 Table).

## Supporting Information

S1 Fig Effects of ethanol on *PRR7* and *PRR9* expression in *Alcpro*:::*LHY* plants, *Alcpro*::*GUS* and wild-type plants. Ethanol (6% v/v) was added to different groups of *ALCPro*::*LHY* plants 2, 6 or 10 hours after dawn, and changes in transcript levels were determined after 2 hours as described for Fig 2. Error bars indicate standard errors from three technical replicates.* indicates p <0.05 and ** p<0.01 as determined by t-tests.
(TIFF)

S2 Fig LHY and CCA1 down-regulate their own and each other’s expression. Expression of the *Alcpro*::*LHY (A-C)* or *Alcpro*::*CCA1* transgenes (D-F) was induced by ethanol (1% v/v) at ZT 17 as described in Fig 2. **(A,D)** show the resulting increases in total LHY protein and *CCA1* mRNA expression, respectively. **(B,E**) show effects of endogenous *LHY* mRNA levels and **(C,F)** on endogenous *CCA*1 mRNA levels. LHY protein levels were quantified as in Fig 2. *LHY* and *CCA1* mRNA levels were assayed by quantitative PCR, normalized to *ACTIN* mRNA and expressed relative to wild-type levels at time zero. Specific amplification of the endogenous *LHY* and *CCA1* mRNAs was achieved using primers to the 5’untranslated region (5’UTR) of the genes. Error bars represent standard errors of the mean from three technical replicates. **(G)** Immunoblot showing changes in LHY protein levels after ethanol addition. The LHY protein is indicated by filled triangles, and a constitutive, cross-reactive band is indicated by open triangles. B indicates bacterially expressed LHY protein. As a loading control, the lower part of the gel was stained with Coomassie blue to reveal the RBCS protein.
(TIFF)

S1 File Numerical data for Figs 1, 2, 3 and S1 and S2 Figs. (XLSX)

S1 Table Oligonucleotide primer sequences. (DOCX)
