# Supplementary figures and images for: Revised Morning Loops of the Arabidopsis Circadian Clock Based on Analyses of Direct Regulatory Interactions
Source: PLoS One. 2015 Dec 1;10(12):e0143943. doi: 10.1371/journal.pone.0143943 (PMC4666590; doi:10.1371/journal.pone.0143943)

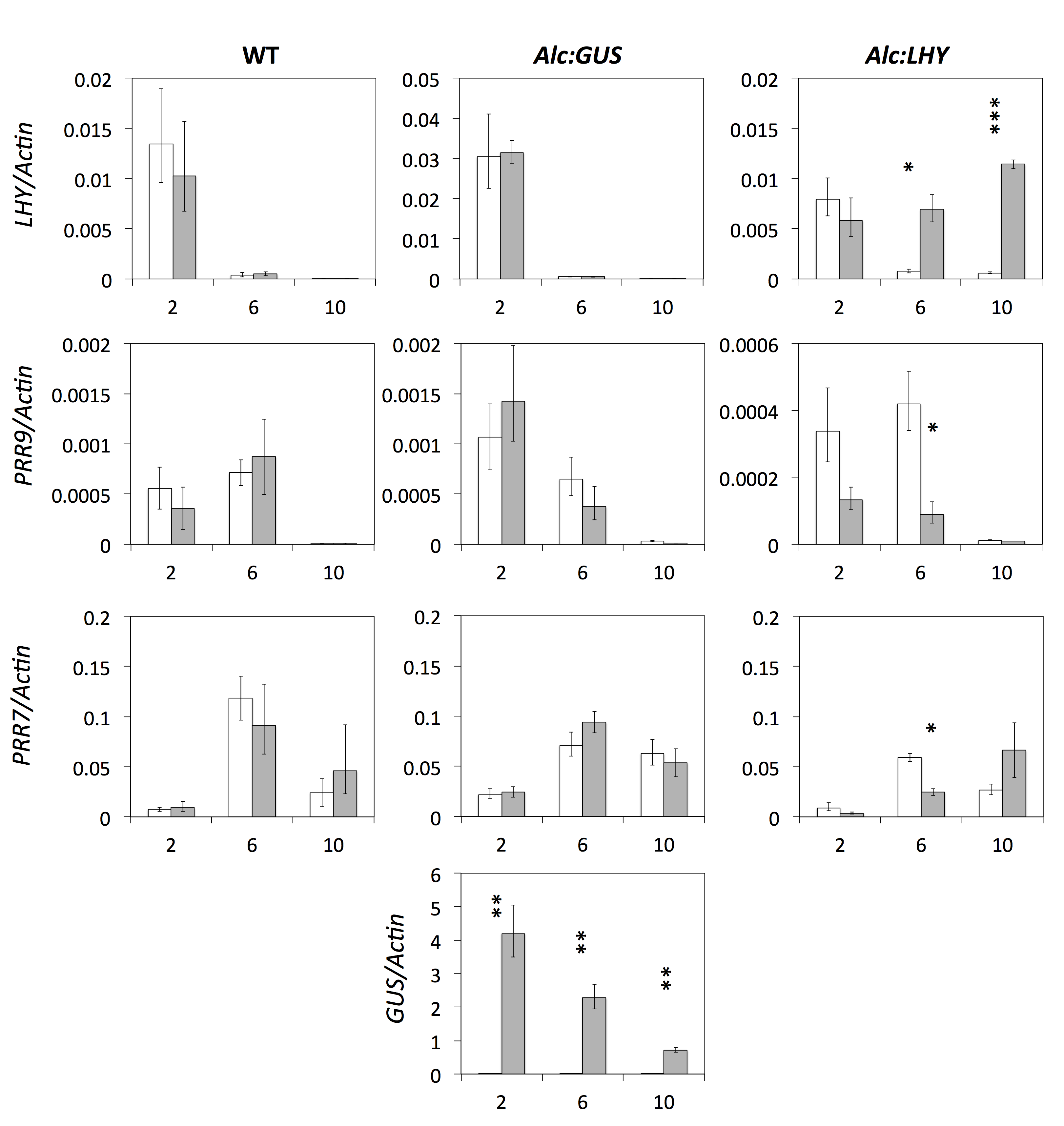

Supplement: S1 Fig — Ethanol (6% v/v) was added to different groups of ALCPro::LHY plants 2, 6 or 10 hours after dawn, and changes in transcript levels were determined after 2 hours as described for Fig 2. Error bars indicate standard errors from three technical replicates.* indicates p <0.05 and ** p<0.01 as determined by t-tests. (TIFF) [file pone.0143943.s001.tiff]

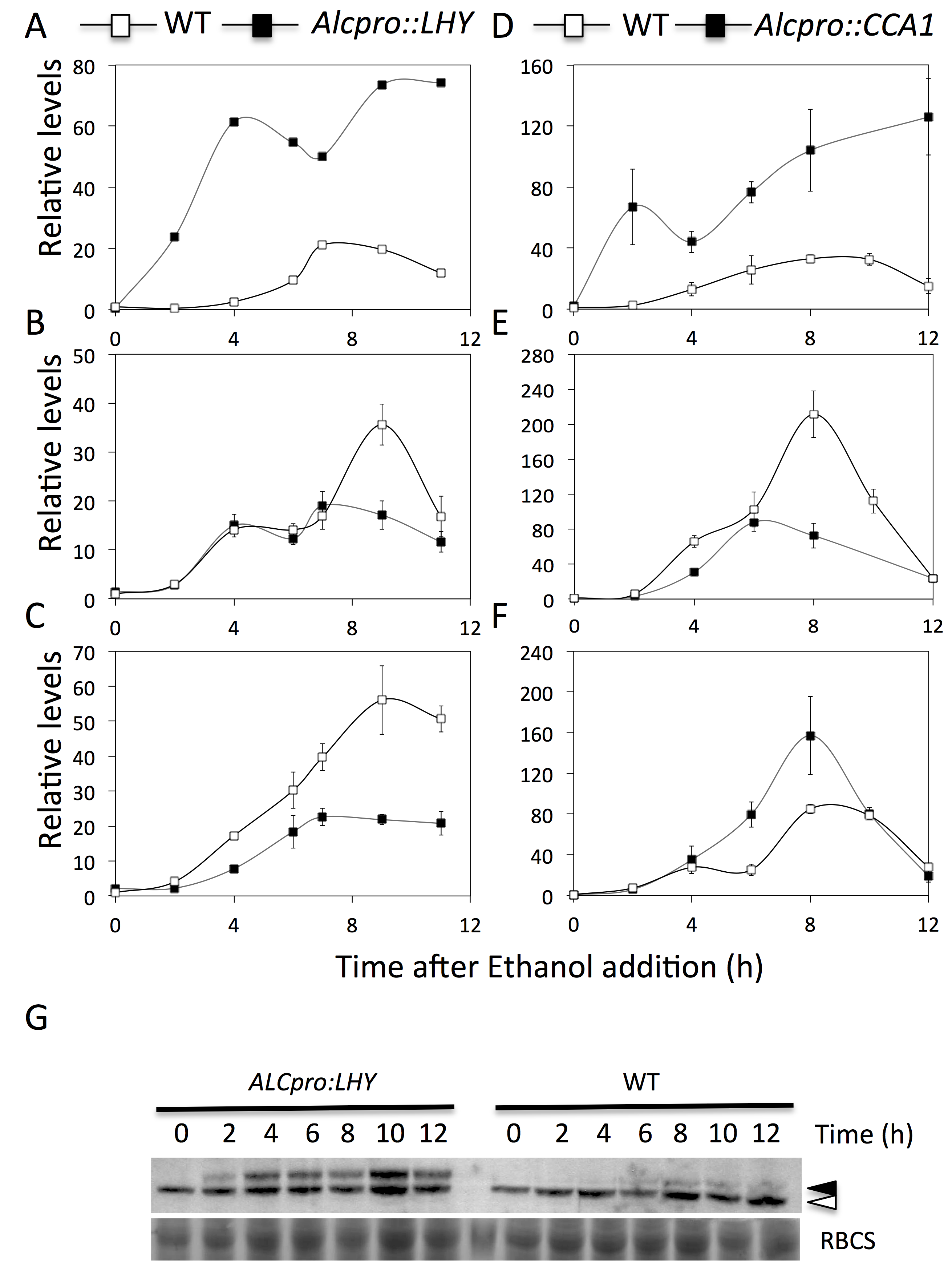

Supplement: S2 Fig — Expression of the Alcpro::LHY (A-C) or Alcpro::CCA1 transgenes (D-F) was induced by ethanol (1% v/v) at ZT 17 as described in Fig 2. (A,D) show the resulting increases in total LHY protein and CCA1 mRNA expression, respectively. (B,E) show effects of endogenous LHY mRNA levels and (C,F) on endogenous CCA1 mRNA levels. LHY protein levels were quantified as in Fig 2. LHY and CCA1 mRNA levels were assayed by quantitative PCR, normalized to ACTIN mRNA and expressed relative to wild-type levels at time zero. Specific amplification of the endogenous LHY and CCA1 mRNAs was achieved using primers to the 5’untranslated region (5’UTR) of the genes. Error bars represent standard errors of the mean from three technical replicates. (G) Immunoblot showing changes in LHY protein levels after ethanol addition. The LHY protein is indicated by filled triangles, and a constitutive, cross-reactive band is indicated by open triangles. B indicates bacterially expressed LHY protein. As a loading control, the lower part of the gel was stained with Coomassie blue to reveal the RBCS protein. (TIFF) [file pone.0143943.s002.tiff]
