# Supplementary material for: Revised Morning Loops of the Arabidopsis Circadian Clock Based on Analyses of Direct Regulatory Interactions
Source: PLoS One. 2015 Dec 1;10(12):e0143943. doi: 10.1371/journal.pone.0143943 (PMC4666590; doi:10.1371/journal.pone.0143943)
Supplement: S1 Table — (DOCX) [file pone.0143943.s004.docx]

**S1 Table: Oligonucleotide primer sequences.**

|  | **Locus** | **Forward primer** | **Reverse primer** |
| --- | --- | --- | --- |
| **(A)** | *ACTIN* *2/7* 3’UTR | GTATCGGGTGACAATGCAGCTATTA | TGCTGGAGTAAAACATAAGCCACTC |
|  | *LHY* promoter | TCGTTTACACGTAAGCAATTGTGG | CAGATCGACACGTGGTGATATTGTA |
|  | *CCA1* promoter | AGAGTGTGAGAATAGCGCGTGTAGT | GATCGTTAATCTCTCCCACCAGTTT |
|  | *TOC1* promoter | TTTGTTGATTTTGATATGGAGATGC | GGTTGTGTTGGATAGTTTGGTTGAG |
|  | *PRR3* promoter | TCAGCATGCGTAAATTTATCTGGTC | GGAGGTGGTAGAACAAAAAGGTCAG |
|  | *PRR5* promoter | TTGCTGATGTGGCAAACGTGGC | GAAAGTGGGTGGCGAACCGACC |
|  | *PRR7* promoter | GCCTCCTGAACGACTGTTTTTG | CAGAGCGGATATTTCCACATCA |
|  | *PRR9* promoter | GCAGAGGACCACCTCCACCGAA | CGGTGTGTAACAAAGCGGGCCT |
|  | *LUX* promoter | CCGGTTTTGCCCACTTTCT | AAGTCAGCTATTTGTGAGGGTTTTTC |
|  | *ELF4* promoter | ACGCGCGTGTTCTTTGATATC | ACCGAGGCGAGTAAGTTCTGTT |
|  | *GI* promoter | GTGTGAGGCCCATTTAGATATTTTC | GCCGTTCGATGGTTGTAGATAAAC |
| **(B)** | *LHY* CDS | GCAAATCTTCAAGCTACAACAGCAT | TATCTGGAGAAACGAACGGTAATCA |
|  | *LHY* 5’UTR | TTTCATGTTTGGGGAGATCAAAGAT | TTGTATATGGCTTTCTTGCCTTAGC |
|  | *CCA1* CDS | CCAAGGTAGAGAAAGAGGCTGAAG | GGCCGTGGAGGAGGAATAG |
|  | *CCA1* 5’UTR | ATCCAAGCTGATTTTGTTTCTTTCA | ATGGCTTCCGAGTCTTAATAACCAG |
| **(C)** | *-957/+1 LHY :luc* transgene | GACTCACTATAGGGCGAATTGGGTA | CTGGAACAGCACCAAGGGTATAAAT |
|  | LHY WT promoter | AGCAATTGTGGACCACCACACTCA | GAGGCTGGAACAGCACCAAGGG |

(A) primers used for analyses of ChIP samples. (B) Primers used for measurement of transcript levels for endogenous and transgenic (ethanol-inducible) copies of the *LHY* and *CCA1* genes.
